# Diagnosis of Leiomyosarcoma after Uterine Artery Embolization for Multiple Leiomyomas

**DOI:** 10.1155/2020/8823428

**Published:** 2020-11-03

**Authors:** Maako Tsuji, Tomoko Kihara, Shuko Hushimi, Yukari Nishino, Tomoko Kanayama, Mitsuhiro Nishijima

**Affiliations:** Department of Obstetrics and Gynecology, Hyogo Prefectural Awaji Medical Center, Japan

## Abstract

Uterine sarcoma is significantly rarer than leiomyoma and has poor prognosis. Moreover, the diagnosis of leiomyosarcoma is difficult because its symptoms, including pelvic pain, uterine mass, and/or uterine bleeding, are very similar to those of leiomyoma. There are a few cases of leiomyosarcoma wherein leiomyoma was treated with uterine artery embolization (UAE); these reports revealed that the symptoms of hypermenorrhea or/and pelvic pain persisted even after UAE. Symptoms persisting even after UAE treatment for leiomyomas, especially multiple leiomyomas, should be investigated to rule out leiomyosarcoma. Therefore, long-term follow-up is needed. Here, we describe a case of a 39-year-old woman diagnosed with leiomyosarcoma 3 years after undergoing UAE for multiple leiomyomas.

## 1. Introduction

Uterine leiomyoma is the most common pelvic tumor in women, and uterine sarcoma is significantly rarer than leiomyomas and has poor prognosis. The clinical features of leiomyomas and uterine sarcomas are often indistinguishable; thus, the diagnosis of uterine sarcoma is challenging.

One of the treatments for leiomyomas is uterine artery embolization (UAE), whereas the first-line treatment for leiomyosarcoma is hysterectomy. If leiomyomas are treated with UAE, they should be reevaluated and followed up over a long period of time, as several case reports describe leiomyosarcomas that were initially incorrectly diagnosed as leiomyomas and treated with UAE. Here, we report about a woman diagnosed with leiomyosarcoma 3 years after UAE for multiple leiomyomas.

## 2. Case Presentation

**Figure 1:**
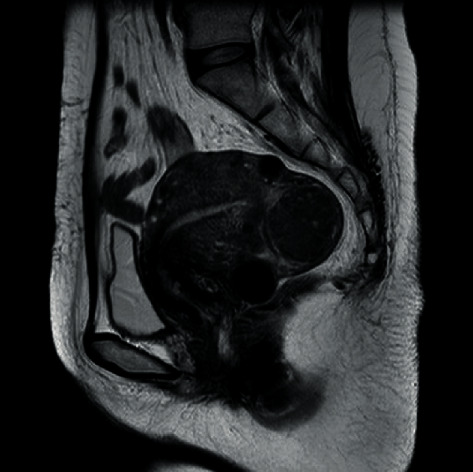
Magnetic resonance imaging was performed before uterine artery embolization. MR images show clearly visible fibroids and not tumors.

A 39-year-old woman, gravida 2, para 2, underwent myomectomy for leiomyomata at 22 years of age and was treated with low-dose oral contraceptives (OCs) for hypermenorrhea at 34 years of age. After 5 years, the leiomyomata relapsed. Magnetic resonance imaging (MRI) showed multiple leiomyomata measuring ~4 cm (Figure 1), and the endometrial sampling was negative. UAE was performed as the patient wished to preserve the uterus.

**Figure 2:**
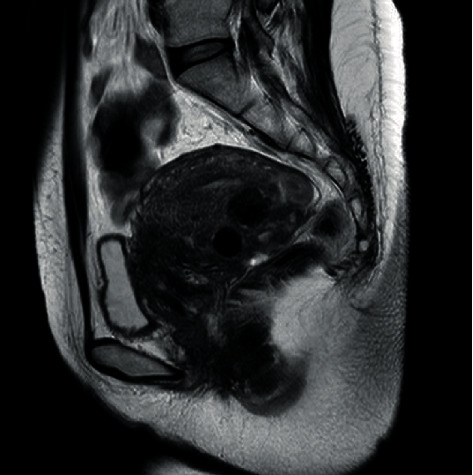
Magnetic resonance imaging was performed 2 years after uterine artery embolization. The leiomyomas grew smaller.

Three months after UAE, the hypermenorrhea improved, and MRI revealed that the uterus mass decreased from 4 cm to 1.7 cm in size. However, she experienced renewed pelvic pain and bleeding. We determined that UAE had an effect on the leiomyoma but not on dysmenorrhea, and OCs were reinitiated. Two years after UAE, the leiomyoma became smaller (Figure 2).

**Figure 3:**
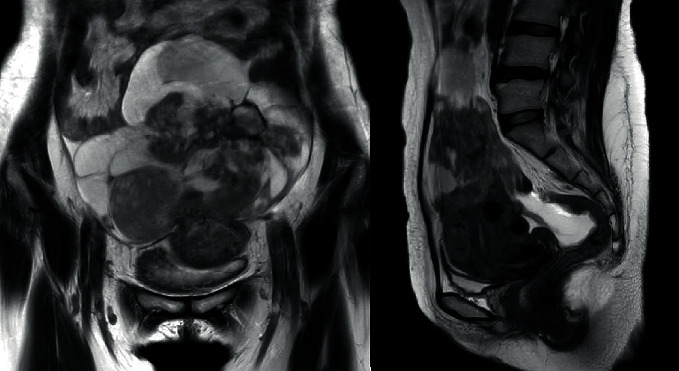
Magnetic resonance imaging was performed 3.5 years after uterine artery embolization. The leiomyomas were stable, and a new uterine mass was noted.

Three and a half years after UAE, the patient returned to the hospital with abdominal pain and bloating. Transvaginal sonography (TVS) showed an enlarged uterus (14 cm) with multiple irregularly shaped hyperechoic lesions. The MRI showed a decrease in the multiple leiomyomas, but myometrial thickening of the uterine fundus was noted, with strong and homogeneous enhancements on gadolinium-enhanced T1/T2-weighted images (Figure 3). It is not clear that she had actually sarcoma from the beginning or developed sarcoma during treatment for leiomyomas. Because sarcoma was suspected, she was referred to a higher-order medical institution. She underwent total abdominal hysterectomy with bilateral oophorectomy, pelvic lymphadenectomy, and omentectomy. The uterus measured 14 × 19 cm. There was a pedunculated neonatal head-sized tumor at the uterine bottom and multiple egg-sized leiomyomas in the uterine body. Final pathology examination results showed leiomyosarcoma with mitotic figures (5/10 HPF), atypia, and tumor cell necrosis. The Ki-67 index was 20%. The patient was discharged from the hospital in good condition. No adjuvant therapy was recommended, and there was no disease evidence during her 6-month postoperative visit.

## 3. Discussion

Uterine sarcoma is a rare, aggressive uterine malignancy associated with high risks of recurrence and death. Most uterine sarcomas occur in women aged >40 years [1, 2]. Endometrial stromal sarcomas and leiomyosarcomas are the two most common uterine sarcoma types.

Leiomyosarcoma diagnosis is difficult due to symptom similarity with leiomyomas. The incidence of uterine sarcoma in patients operated on for presumed leiomyomas is approximately 0.24% [3]. Although sarcoma should be suspected in women with a rapid growth of uterus or leiomyoma, data do not support increased risks of malignant neoplasm in such patients or in those with large uterine size [4]. In premenopausal women with presumed uterine leiomyomas, uterine sarcoma diagnosis is considered if bleeding is disproportionate to the uterine size and the patient reports significant pain. Some cases are diagnosed preoperatively based on endometrial sampling, but the positive predictive value is only 52% [5]. MRI may be helpful in women suspected to have sarcoma; however, it does not provide a definitive diagnosis. Uterine sarcoma diagnosis is based on histologic examination [6, 7]. Leiomyosarcomas have mitotic index, cellular atypia, and geographic areas of coagulative necrosis separated from viable neoplasm.

UAE is the treatment of choice for symptomatic leiomyoma. UAE is a low cost and invasive treatment compared with myomectomy. Van der Kooij et al. [8] reported that the uterus volume in the patients undergoing UAE decreased by 50-60%, and the symptoms (e.g., uterine bleeding and pelvic pain) in such patients improved by 82.7% after a year and 85.3% after 5 years.

We reviewed 10 case reports of leiomyosarcoma diagnosed after UAE (Table 1). These reports were identified using search engines, e.g., PubMed, for “sarcoma” and “UAE.” In the table, 6 patients were observed having single leiomyoma, 2 patients were having multiple leiomyomas, and 2 patients were having unknown number of leiomyomas. Unexpectedly, increased tumor size was noted in 2 patients, whereas temporarily unchanged or decreased tumor size was noted in 5 patients. These changes were because the tumors had reduced blood supply for a small interval of time and then had started to grow again a few months after the procedure [3]. Conversely, the symptoms of 4 patients were stable and those of 5 patients reappeared after a few months––a time period shorter than the time for regrowth of uterine volume after UAE. The number of case reports was small and shown for reference only; however, almost all symptoms of leiomyomas improved after UAE. Taking into consideration for almost symptoms of leiomyoma improved after UAE [8], persisting symptoms even after UAE to treat leiomyoma suggests a possibility of leiomyosarcoma.

In this case, the patient's uterus volume decreased for 2 years after UAE, but the symptoms persisted. It is not clear that the patient had actually sarcoma from the beginning; however, it is difficult to rediagnose sarcoma, once diagnosed with being not a sarcoma but leiomyoma based on endometrial sampling, MRI, and so on. We must consider the possibility to misdiagnose sarcoma as leiomyoma or to develop sarcoma during treatment for leiomyoma.

In conclusion, it is important to detect and treat leiomyosarcomas early because they are aggressive tumors. Leiomyosarcoma and leiomyoma have similar symptoms; thus, we must be careful of misdiagnosing leiomyosarcoma. Symptoms persisting after using UAE to treat leiomyomas should be investigated to rule out sarcoma. Hence, long-term follow-ups are needed.

## Figures and Tables

**Table 1:** Summary of leiomyosarcoma cases diagnosed after uterine artery embolization treatment for leiomyoma.

Case	Age	Size (single or multiple)	Symptom onset after UAE	Size after UAE	Diagnosis after UAE	Opportunity of diagnosis
Al-Badr and Faught [9]	52	7 (single)			1 day	
Joyce et al. [10]	51	—	Stable		1 month	Stable symptom
Common et al. [11]	49	8 (single)	Stable	Regrowth	6 months	Regrowth
D'Angelo et al. [12]	41	10 (single)	12 months	Smaller	15 months	Regrowth after 15 months
Goldberg et al. [13]	45	9/4 (multiple)	13 months	Stable	13 months	Returned symptom
Papadia and Salom [3]	36	9 (single)	8 months	Regrowth	14 months	Regrowth
Choi et al. [14]	36	11 (single)	3 months	Smaller after 3 months	4 months	Suspicion of malignancy for imaging
Kainsbaka et al. [15]	—	6 (single)	Stable	Smaller after 6 months	9 months	Suspicion of malignancy for imaging
Posy et al. [16]	50	—	Stable	Stable	5 years	Lung metastasis
Buzaglo et al. [17]	45	9 (multiple)	6 months	Smaller after 6 months	6 months	Suspicion of malignancy for exploratory laparotomy
